# Palmitic acid-induced cell death: impact of endoplasmic reticulum and oxidative stress, mitigated by L-citrulline

**DOI:** 10.5713/ab.24.0249

**Published:** 2024-08-26

**Authors:** Md. Rezwanul Habib, Yukako Tokutake, Shinichi Yonekura

**Affiliations:** 1 Graduate School of Medicine, Science and Technology, Shinshu University, Nagano 399-4598, Japan; 2 Institute of Agriculture, Academic Assembly, Shinshu University, Nagano 399-4598, Japan

**Keywords:** Bovine Mammary Epithelial Cell, Endoplasmic Reticulum Stress, L-citrulline, Oxidative Stress

## Abstract

**Objective:**

Palmitic acid (PA), the most abundant saturated free fatty acids, induces apoptosis in bovine mammary epithelial cells (MECs). It is suggested that oxidative stress and endoplasmic reticulum (ER) stress are key mechanisms underlying PA-induced cell death. This study aimed to investigate the interaction between ER stress and oxidative stress during PA-induced cell death in mammary alveolar cell-T (MAC-T) cells. Additionally, we examined whether L-citrulline can protect against PA-induced damage of MAC-T cells.

**Methods:**

MAC-T cells were treated with 4-phenyl butyric acid (4-PBA) or N-acetyl-L-cysteine (NAC) to inhibit PA-induced ER stress and oxidative stress, respectively. MAC-T cells were pretreated with or without L-citrulline for 48 h followed by PA treatment. Cell viability was measured with MTT assays. Intracellular reactive oxygen species (ROS) levels in MAC-T cells were assessed using 5-(and-6)-chloromethyl- 2′,7′-dichlorodihydrofluorescein diacetate acetyl ester dye. Real-time quantitative polymerase chain reaction was used to explore the regulation of genes associated with oxidative stress, and ER stress genes. Western blotting analysis was also carried out.

**Results:**

4-PBA significantly reduced PA-induced mRNA expressions of activating transcription factor 4 (*ATF4*), C/EBP homologous protein (*CHOP*), nuclear factor (erythroid-derived 2)-like 2 (*NRF2*), and intracellular ROS levels. Furthermore, NAC dramatically reduced PA-induced ROS levels and the mRNA expressions of *NRF2*, *ATF4*, and *CHOP*. L-citrulline pretreatment effectively rescued cell viability decreased by PA. Moreover, L-citrulline pretreatment significantly downregulated the PA-induced upregulation of *GRP78*, *ATF4*, and *CHOP* mRNA expression, and protein expression of p-PERK and cleaved caspase-3. PA increased intracellular ROS levels and *NRF2* mRNA expression, whereas L-citrulline pretreatment remarkably reduced these levels.

**Conclusion:**

Both ER and oxidative stresses interact during PA-induced cell death in MAC-T cells, and L-citrulline could attenuate this cell death by inhibiting ER and oxidative stresses. Therefore, L-citrulline may be a promising supplement for protecting against PA-induced cell death in bovine MECs during the lactation period of dairy cows.

## INTRODUCTION

Mammary glands consist of millions of secretory cells, known as mammary epithelial cells (MECs), which are crucial for milk production. The quantity and secretory activity of MECs determine the volume of milk produced during lactation [1], making their preservation vital for optimal milk production.

Over the past decade, global milk production has doubled due to intensive breeding strategies, improved nutrition, and better management of high-yielding dairy animals [2]. While economically beneficial, these advancements have led to some negative consequences. For instance, the rapid increase in milk yield and milk component synthesis at the onset of lactation [3], coupled with a higher nutrient demand, results in a negative energy balance (NEB) [4], a leading cause of reduced milk yield.

Under NEB conditions, dairy cows metabolize body fat to meet energy requirements, producing non-esterified fatty acids (NEFAs) [4]. Palmitic acid (PA), the most abundant saturated NEFA (comprising 34% of total NEFAs), is utilized by MECs as an energy substrate and for milk lipid synthesis [5]. However, numerous studies have shown that PA induces apoptosis in various cells, including neurons [6], and endothelial cells [7]. Our previous research also found that PA triggers endoplasmic reticulum (ER) stress-mediated apoptosis in bovine MECs [8], making PA significant factor in reducing the number of MECs and milk yield during early lactation.

When proteins in the ER are misfolded or improperly folded, it can induce ER stress. In mild stress conditions, the PKR-like ER kinase (PERK) protein is activated, inhibiting the translation of general proteins and promoting the translation of activating transcription factor 4 (*ATF4*) by phosphorylating eukaryotic initiation factor 2α [9]. Under extreme or prolonged ER stress conditions, *ATF4* promotes the transcription of C/EBP homologous protein (*CHOP*), a transcription factor that triggers cell apoptosis [10]. Previous studies have reported that CHOP triggers PA-induced apoptosis in Saos-2 cells [11] and H9c2 cells [12]. In addition to ER stress, oxidative stress also important for PA-induced cell death. In pancreatic β-cells [13,14], PA induces apoptosis through oxidative stress mediation. Multiple reports suggest that both ER stress and oxidative stress are key mechanisms of PA-induced cell death. Recent evidence suggests that ER stress is linked to the generation of reactive oxygen species (ROS, markers of oxidative stress) [15]. On the contrary, the overproduction of ROS induces ER stress through misfolding of protein in the ER, cause cell apoptosis [15].

However, it remains unknown which one, either ER stress or oxidative stress is predominant in PA-induced cell death in mammary alveolar cell-T (MAC-T) cells. Therefore, this study aimed to address this gap and gain insights into cell specific mechanisms through investigating the interplay between ER stress and oxidative stress during PA-induced apoptosis in MAC-T cells.

L-citrulline is a non-essential alpha-amino acid found in various fruits and vegetables, with watermelon being the primary dietary source of L-citrulline [16], and endogenous synthesis is the principal source in the body [17]. L-citrulline plays a vital role in cellular metabolism and organ function in most living systems [17]. Previous studies have shown that L-citrulline acts as an effective scavenger of ROS-mediated endothelial dysfunction [18], prevents inflammation and oxidative stress-induced skeletal muscle cell wasting [19], and protects against heat-induced mitochondrial dysfunction and cell injury [20]. However, it is currently unknown whether L-citrulline offers any protective benefits against PA-induced cell death in MAC-T cells.

Therefore, this study aimed to investigate the interaction between ER stress and oxidative stress using an ER stress inhibitor and an ROS scavenger during PA-induced cell death in MAC-T cells (a bovine MEC line stably transfected with the large T-antigen of simian vacuolating virus 40). Additionally, we examined whether L-citrulline can protect against PA-induced cell damage in cultured MAC-T cells by inhibiting ER and oxidative stresses. The viability of MAC-T cells was assessed after pretreatment with L-citrulline followed by PA treatment. Furthermore, ER and oxidative stress-related genes and intracellular ROS levels were examined in PA-treated MAC-T cells with or without L-citrulline pretreatment.

## MATERIALS AND METHODS

### Reagents and chemicals

Dulbecco’s modified eagle medium (DMEM; cat. # D7777), bovine hydrocortisone (cat. # H0888), insulin solution from bovine pancreas (cat. # I0516) and N-acetyl-L-cysteine (NAC, cat. # A7250) were purchased from Sigma-Aldrich (St. Louis, MO, USA). Fetal bovine serum (FBS; cat. # SH30396.03) was obtained from Hyclone Laboratories (Logan, UT, USA). Penicillin-streptomycin mixed solution (cat. # 09367-34), L-trypsin (cat. # 32778-05), sodium hydrogen carbonate (cat. # 31213-15), and palmitic acid (cat. # 25918-72) were purchased from Nacalai Tesque (Kyoto, Japan). Albumin solution (30% w/v) from bovine serum albumin (BSA) (cat. # 017-22231), L-citrulline (cat. # 036-21402), methylcellulose 100 (cat. # 133-07182) and dimethyl sulfoxide (cat. # 048-21985) were obtained from Fujifilm Wako Pure Chemical Corporation (Osaka, Japan). Sodium 4-phenylbutyrate (4-PBA, cat. # 1716-12-7) was purchased from Tokyo Chemical Industry Co., Ltd. (Tokyo, Japan).

### Preparation of palmitic acid and L-citrulline solutions

The stock solutions of PA and L-citrulline were prepared by dissolving the required amounts of PA and L-citrulline into dimethyl sulfoxide and methylcellulose solutions (0.25%), respectively. Both solutions were heated to 55°C in a water bath with continuous shaking. The desired concentration of PA stock solution was achieved by mixing 10% (wt/vol) BSA–PBS (fatty acid-free) and warming at 55°C for 15 to 20 min. After that, PA and L-citrulline solutions were filtered using a sterile syringe filter (0.20 μm) and stored at −20°C. Before application to the cells, these solutions were added to the cell culture medium (DMEM supplemented with 10% FBS) to obtain the desired final concentrations.

### Cell culture and treatment

The immortalized bovine mammary epithelial cell line (MAC-T cells) was generously provided by Sangun Roh (Tohoku University, Sendai, Japan). The MAC-T cells were cultured in DMEM supplemented with 10% FBS, bovine insulin (5 μg/mL), streptomycin and penicillin (1%), and hydrocortisone (1 μg/mL). L-citrulline was added to MAC-T cells 48 h prior to PA treatment to evaluate its effectiveness in preventing PA-related damage. All cells were incubated at 37°C in a 5% CO_2_ incubator.

### RNA extraction and quantitative real-time polymerase chain reaction

Total RNA was isolated from MAC-T cells using Sepasol-RNA I Super G (Nacalai Tesque Inc., Japan) according to the manufacturer’s protocol. The concentration and purity of the isolated RNA were determined by optical density measurements at 260 nm and the 260/280 nm wavelength ratio, respectively (NanoDrop One Spectrophotometer; Thermo Fisher Scientific, Waltham, MA, USA). The cDNA was synthesized from total RNA using a qPCR-RT Master Mix with gDNA Remover (TOYOBO, Osaka, Japan). The quantitative real-time (qRT) polymerase chain reaction (PCR) assays were performed using the SYBR Premix Ex Taq TM II (TaKaRa Bio Inc., Shiga, Japan) using the StepOne Real-Time PCR System (Applied Biosystems, Waltham, MA, USA). The specific primers used in the quantitative PCR are mentioned in Table 1. The relative expression of each target gene was normalized to *ACTB* (β-actin) and calculated using the 2^−ΔΔCt^ method [23]. The mRNA expression values were represented as relative values to the control.

### Analysis of intracellular reactive oxygen species production

Intracellular ROS levels in MAC-T cells were assessed using 5-(and-6)-chloromethyl- 2′,7′-dichlorodihydrofluorescein diacetate acetyl ester (CM-H_2_DCFDA) dye (Invitrogen, Waltham, MA, USA) following the manufacturer’s protocol. MAC-T cells were cultured in a 24-well plate and pretreated with L-citrulline or without pretreatment with 4-PBA and NAC. Confluent cells (90% to 100%) were then treated with various concentrations of L-citrulline (25, 50, and 100 μM), 4-PBA (300 μM), and NAC (1 mM), followed by a PA challenge for 24 h. After treatment, cells were washed with phosphate buffered saline solution (1×PBS) and loaded with 5 μM CM-H_2_DCFDA diluted in PBS. Following a 30 min incubation at 37°C in the dark, the ROS dye was removed, and cells were washed three times with PBS to eliminate any unincorporated dye. Subsequently, a prewarmed growth medium was added to each well, and green fluorescence microphotographs were captured using an EVOS FL auto-imaging system (Thermo Fisher Scientific, USA). All dichlorofluorescein (DCF) fluorescence intensities were background subtracted and normalized to the background-subtracted control value [24] using Image J software (National Institutes of Health, Bethesda, MD, USA). Ten views were analyzed for each experiment to determine the fluorescence intensity, and data from three independent experiments were combined, analyzed, and expressed as fold of control.

### Cell viability and L-citrulline toxicity determination

Cell viability and L-citrulline toxicity assessments were conducted using the 3-(4,5-dimethylthiazol-2-yl)-2,5-diphenyltetrazolium bromide (MTT) cell viability assay kit (Biotium, Fremont, CA, USA) following the manufacturer’s instructions. MAC-T cells were seeded at a density of 0.5×10^4^ cells/well in a 96-well plate and incubated at 37°C in a 5% CO_2_ incubator for 48 h to reach 90% to 100% confluency. Various concentrations of L-citrulline (0, 25, 50, and 100 μM) were added to the cells and incubated for 48 h to assess L-citrulline toxicity. MAC-T cells were pretreated with L-citrulline (25, 50, and 100 μM) followed by PA challenge for 24 h to examine the effects of L-citrulline on cellular damage induced by PA. After the incubation period, 10 μL MTT solution was added to each well (containing 100 μL of cell culture medium) and incubated at 37°C for 4 h, followed by the addition of 200 μL of dimethyl sulfoxide. Absorbance was measured using a Multiskan SkyHigh absorbance microplate reader (Thermo Fisher Scientific, USA) at 570 nm with a reference wavelength of 630 nm.

### Protein extraction and Western blotting

Cells were washed two times and then lysed using a radio-immunoprecipitation assay (RIPA) lysis buffer (50 mM Tris-HCl pH 7.4, 150 mM NaCl, 0.05% sodium dodecyl sulfate [SDS], 0.2% sodium deoxycholate, 1 mM ethylenediaminetetraacetic acid, and 1% Tergitol-type NP-40) with protease inhibitor cocktail (Nacalai Tesque, Japan). The lysates were then incubated on ice for 30 min. After centrifugation (10 min at 20,000×g), the protein concentrations in the lysates were measured using the Bio-Rad protein assay kit (Bio-Rad Laboratories, Hercules, CA, USA). The cell extracts (40 μg) were run on a 4% to 20% polyacrylamide gel using SDS-PAGE, after which they were transferred onto a polyvinylidene difluoride (PVDF) membrane. The membrane was subsequently placed in a blocking buffer consisting of 0.1% Tween 20 in PBS with 4% skim milk powder and incubated for 1 h. After that, membranes were incubated with an anti-phosphorylated PERK (Santa Cruz Biotechnology, Dallas, TX, USA), anti-PERK (Santa Cruz Biotechnology, USA), anti-cleaved caspase-3 (Cell Signaling Technology, Danvers, MA, USA), and anti-α-tubulin (MBL Co., Nagoya, Japan) antibody diluted in blocking buffer at room temperature. The membranes were then incubated with an anti-rabbit IgG secondary antibody (GE Healthcare, Pittsburgh, PA, USA). Labeled proteins were visualized using the ECL (Enhanced Chemiluminescent) Prime Western Blotting Detection Reagent kit (GE Healthcare, USA) and images were captured using an Image Quant LAS 500 (GE Healthcare, USA). Captured images were analyzed with the Image J software (National Institutes of Health, USA).

### Statistical analysis

The data were expressed as means±standard error of the mean from a typical experiment which was run in triplicates dishes and analyzed using SPSS Software (Version 20.0; IBM Corp., Armonk, NY, USA). Statistical analysis was conducted using a one-way analysis of variance followed by Tukey’s honestly significant difference test for multiple comparisons. Differences with a p-value<0.05 were considered statistically significant.

## RESULTS

### Endoplasmic reticulum stress mediates palmitic acid-induced oxidative stress in MAC-T cells

**Figure 1:**
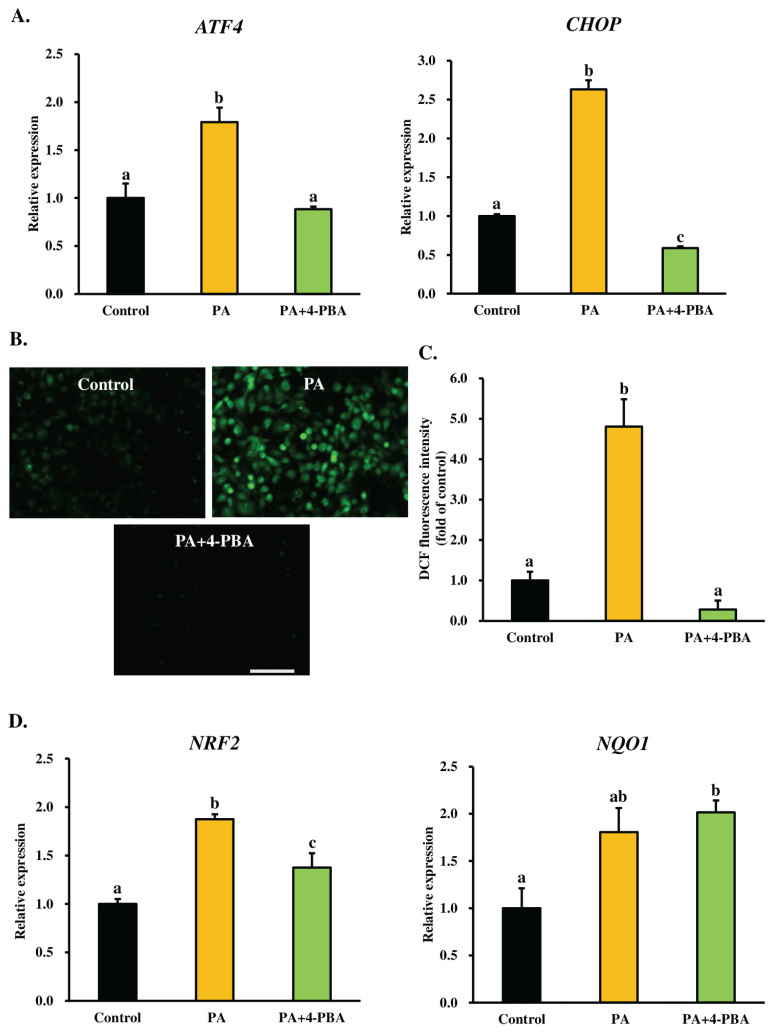
Endoplasmic reticulum (ER) stress mediates PA-induced oxidative stress in MAC-T cells. Confluent cells (90% to 100%) were treated with 300 μM PA, either alone or in combination with 4-PBA, 300 μM), for a duration of 24 h. (A) The mRNA levels of ER stress markers *ATF4* and *CHOP* were determined using RT-qPCR and normalized to *ACTB* levels. (B) Representative photomicrographs depict CM-H_2_DCFDA (DCF) fluorescence in control cells, cells treated with PA alone, and cells co-treated with 4-PBA and PA. Scale bar = 200 μm. (C) The quantitative analysis of DCF fluorescence is based on the images in Figure 1B. The average fluorescence intensity was obtained from ten images for each experiment. The data from three independent experiments were combined, analyzed, and expressed as the fold change relative to control cells, presented as the mean±SEM. (D) The relative mRNA expressions of *NRF2* and *NQO1* were determined by RT-qPCR and normalized to *ACTB* levels. The relative transcript expression was calculated using the 2^−ΔΔCt^ method and is presented as values relative to the control cells (received no PA and 4-PBA). The data, presented as mean±SEM, are based on three independent experiments. ER, endoplasmic reticulum; PA, palmitic acid; MAC-T, mammary alveolar cell-T; 4-PBA, 4-phenylbutyric acid; *ATF4*, activating transcription factor 4; *CHOP*, C/EBP homologous protein; RT-qPCR, real-time quantitative polymerase chain reaction; *ACTB*, β-actin; CM-H_2_DCFDA, 5-(and-6)-chloromethyl- 2′,7′-dichlorodihydrofluorescein diacetate acetyl ester; DCF, dichlorofluorescein; SEM, standard error of the mean; *NRF2*, nuclear factor (erythroid-derived 2)-like 2; *NQO1*, NAD(P)H quinone oxidoreductase 1. A Tukey’s honestly significant difference test was performed to compare the treatment means. ^a–c^ Means with uncommon letters differed significantly (p<0.05).

Previous studies have indicated that PA induces severe ER stress and oxidative stress-mediated apoptosis in cells [8,13, 14], yet the interaction between ER stress and oxidative stress during PA-induced cell death remains unknown. We hypothesized that ER stress might induce oxidative stress and PA-induced apoptosis in MAC-T cells. To investigate this, we used 4-PBA (300 μM) in MAC-T cells to inhibit PA-induced ER stress during treatments. The 4-PBA is a highly effective ER stress inhibitor utilized to promote protein folding and inhibit the aggregation of misfolded proteins in the ER. Our experiments demonstrated that the mRNA levels of ER stress markers *ATF4* (a master transcription factor that induces the expression of *CHOP*) and *CHOP* (key signaling component involved in ER stress-induced apoptosis) were significantly reduced in the co-treatment with 4-PBA and PA groups compared to the PA-treated group alone (Figure 1A). These findings suggest that 4-PBA may mitigate PA-induced ER stress in MAC-T cells. We further explored the relationship between PA-induced ER stress and oxidative stress by examining intracellular ROS levels and the mRNA expression of oxidative stress-related genes following treatment with PA in the presence or absence of 4-PBA. Our results revealed that cotreatment with 4-PBA and PA markedly decreased PA-induced ROS generation (Figure 1B and 1C) and nuclear factor (erythroid-derived 2)-like 2 (*NRF2*, a master regulator of oxidative stress) mRNA expression (Figure 1D), indicating that PA-induced oxidative stress may be dependent on ER stress. These observations suggest that ER stress mediates PA-induced oxidative stress in MAC-T cells.

### Oxidative stress mediates palmitic acid-induced endoplasmic reticulum stress in MAC-T cells

**Figure 2:**
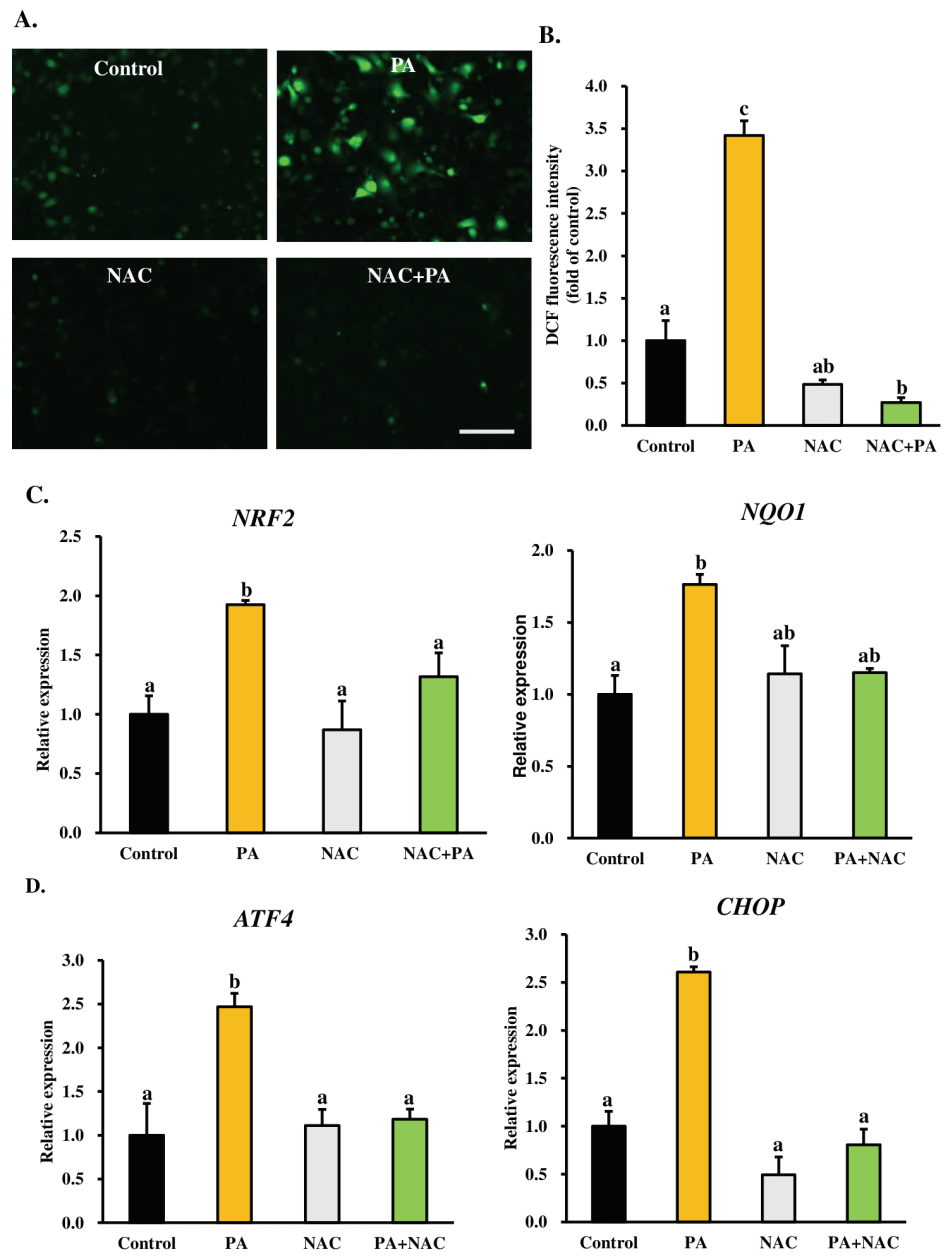
Oxidative stress mediates PA-induced ER stress in MAC-T cells. Confluent MAC-T cells (90% to 100%) were treated with 300 μM PA with or without N-acetyl-L-cysteine (NAC, 1 mM) for 24 h. (A) Representative photomicrographs of DCF fluorescence in control, PA alone, NAC and co-treatment with PA and NAC-treated MAC-T cells. Scale bar = 200 μm. (B) Quantitative analysis of DCF fluorescence analysis based on the images in Figure 2A. Average fluorescence intensity was obtained from ten images for each experiment, and data from three independent experiments were combined, analyzed, and expressed as the fold change relative to control cells, presented as mean±SEM. (C) Relative mRNA expressions of *NRF2* and *NQO1* were determined by RT-qPCR and normalized to *ACTB* levels. (D) mRNA levels of the ER stress markers *ATF4* and *CHOP* were assessed by RT-qPCR and normalized to *ACTB* levels. Relative transcript expression was calculated using the 2^−ΔΔCt^ method and presented as values relative to the control cells (received no PA and NAC). The data are presented as the mean±SEM for three independent experiments. PA, palmitic acid; ER, endoplasmic reticulum; MAC-T, mammary alveolar cell-T; DCF, dichlorofluorescein; SEM, standard error of the mean; RT-qPCR, real-time quantitative polymerase chain reaction; *NRF2*, nuclear factor (erythroid-derived 2)-like 2; *NQO1*, NAD(P)H quinone oxidoreductase 1; *ACTB*, β-actin; *ATF4*, activating transcription factor 4; *CHOP*, C/EBP homologous protein. A Tukey’s honestly significant difference test was conducted to compare treatment means. ^a–c^ Means with uncommon letters differed significantly (p<0.05).

We hypothesized that oxidative stress, induced by PA, is the underlying cause of ER stress-mediated apoptosis in MAC-T cells. To test this, we examined the impact of NAC (a well-established antioxidant) on the ER stress response in cells exposed to PA. NAC, often used to supply cells with sulfhydryl groups, acts as an acetylated precursor of reduced glutathione, and directly interacts with ROS, scavenging oxygen-free radicals [25]. MAC-T cells at confluence (90% to 100%) were treated with 300 μM PA, either alone or in combination with 1 mM NAC, for 24 h. As anticipated, ROS levels significantly decreased in the group treated with NAC alone (0.48± 0.05-fold of control) or in combination with PA (0.27±0.06-fold of control) compared to the group treated with PA alone (3.42±0.17-fold of control) (Figure 2A and 2B). As depicted in Figure 2C, both NAC treatment and co-treatment with NAC and PA significantly reduced *NRF2* mRNA expression compared to the group treated with PA alone. These results suggest that PA induces oxidative stress in MAC-T cells through ROS overproduction, and *NRF2* is activated under PA-induced oxidative stress. Conversely, NAC reduced PA-induced oxidative stress in MAC-T cells. Next, to determine whether PA-induced ER stress is linked to oxidative stress, we examined the effect of NAC on the ER stress markers *ATF4* and *CHOP* mRNA expressions. NAC treatment significantly downregulated the PA-induced upregulation of *ATF4* and *CHOP* mRNA expressions (Figure 2D), suggesting that PA-induced ER stress in MAC-T cells is dependent on oxidative stress.

### L-citrulline attenuated palmitic acid-induced cell viability loss

**Figure 3:**
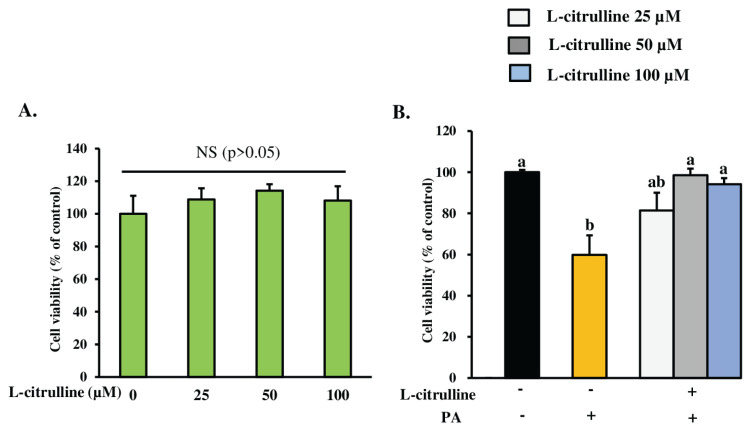
L-citrulline on PA-reduced cell viability in MAC-T cells. (A) Effect of different concentrations of L-citrulline (25, 50, and 100 μM) on the viability of MAC-T cells. The cells, once they reached confluence (90% to 100%), were treated with these concentrations of L-citrulline for 48 h. Cell viability was measured using a MTT assay. Absorbance was measured at 570 and 630 nm using a Multiskan SkyHigh absorbance microplate reader. Survival rates were calculated and expressed as a percentage of control cells (received no L-citrulline). (B) Confluent MAC-T cells (90% to 100%) were treated with 300 μM PA, either alone or with L-citrulline pretreatment at concentrations of 25, 50, and 100 μM, for 24 h. Cell viability was measured using the MTT assay. Absorbance was measured at 570 and 630 nm using a Multiskan SkyHigh absorbance microplate reader. Survival rates were calculated and expressed as a percentage of control cells (received no L-citrulline and PA). The data, presented as mean± standard error of the mean, are based on three independent experiments. PA, palmitic acid; MAC-T, mammary alveolar cell-T; MTT, 3-(4,5-dimethylthiazol-2-yl)-2,5-diphenyltetrazolium bromide; NS, non-significant. A Tukey’s honestly significant difference test was performed to compare the treatment means. ^a,b^ Means with uncommon letters differed significantly (p<0.05).

To evaluate the potential impact of L-citrulline on the viability of MAC-T cells, we treated the cells with three different concentrations (25, 50, and 100 μM) of L-citrulline and assessed the cell viability using the MTT assay. L-citrulline did not significantly (p>0.05) affect cell viability compared to the control group (Figure 3A), indicating that these concentrations of L-citrulline are not toxic to MAC-T cells. Our previous research showed that PA significantly reduced MEC viability [8]. We then examined the effect of L-citrulline pretreatment on changes in cell viability following PA treatment (300 μM) for 24 h. As shown in Figure 3B, L-citrulline pretreatment (50 and 100 μM) significantly rescued cell viability (98%±3.16% and 94%±2.96%) compared with 60% ±9.59% in the PA group from PA-induced reduction. These findings suggest that L-citrulline can effectively mitigate the damage to MAC-T cells induced by PA.

### L-citrulline reduced palmitic acid-induced endoplasmic reticulum stress

**Figure 4:**
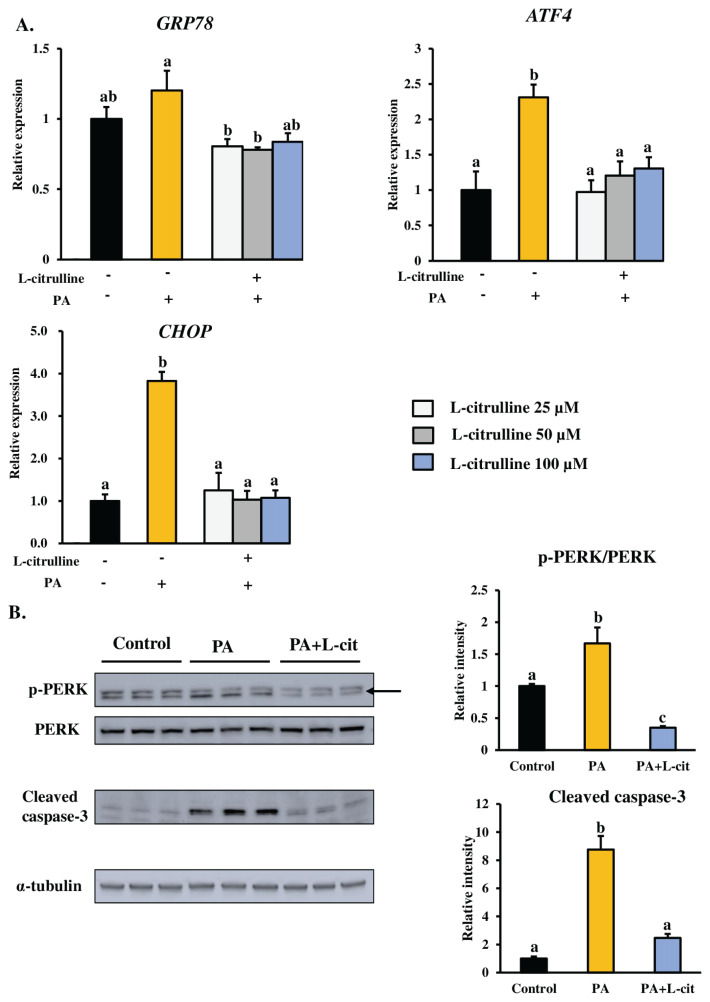
L-citrulline reduced PA-induced ER stress in MAC-T cells. (A) Confluent cells (90% to 100%) were treated with 300 μM PA, either alone or with L-citrulline pretreatment at concentrations of 25, 50, and 100 μM, for 24 h. The relative mRNA expressions of ER stress markers *GRP78*, *ATF4*, and *CHOP* were assessed by RT-qPCR and normalized to *ACTB* levels. The relative transcript expression was calculated using the 2^−ΔΔCt^ method and is presented as values relative to the control cells (received no L-citrulline and PA). The data, presented as the mean±standard error of the mean, are based on three independent experiments. (B) MAC-T cells were treated with 400 μM PA with or without L-citrulline pretreatment at 100 μM, for 12 h, and phospho-PERK, PERK, cleaved caspase-3, and α-tubulin (internal control) protein levels were detected using Western blotting. Left panel: Representative images of three independent experiments are shown. An arrow indicates the reduction of p-PERK. Right panel: Quantification of phospho-PERK/PERK and cleaved caspase-3 expression of three independent experiments and normalized to α-tubulin and represented in the bar graph. PA, palmitic acid; L-cit, L-citrulline; ER, endoplasmic reticulum; MAC-T, mammary alveolar cell-T; *GRP78*, glucose-regulated protein 78; *ATF4*, activating transcription factor 4; *CHOP*, C/EBP homologous protein; RT-qPCR, real-time quantitative polymerase chain reaction; *ACTB*, β-actin; PERK, protein kinase R (PKR)-like endoplasmic reticulum kinase; p-PERK, phosphorylated-PERK. A Tukey’s honestly significant difference test was performed to compare the treatment means. ^a–c^ Means with uncommon letters differed significantly (p<0.05).

Our prior research demonstrated that PA reduces cell numbers via ER stress-induced apoptosis in bovine MECs [8]. To investigate whether L-citrulline pretreatment following PA treatment can mitigate PA-induced ER stress in MAC-T cells, we analyzed *GRP78*, *ATF4*, and *CHOP* as ER stress markers. The results revealed that L-citrulline significantly downregulated the PA-induced upregulation of *GRP78*, *ATF4*, and *CHOP* mRNA expression levels (Figure 4A). Moreover, we performed Western blot analysis to measure phospho-PERK and cleaved caspase-3 protein expression, to confirm the advantageous effects of L-citrulline. As shown in Figure 4B, L-citrulline pretreatment significantly suppressed the PA-induced phospho-PERK and cleaved caspase-3 protein expression. Therefore, these findings suggesting that L-citrulline alleviates PA-induced ER stress in MAC-T cells.

### L-citrulline diminished palmitic acid-induced reactive oxygen species accumulation and alleviated oxidative stress

**Figure 5:**
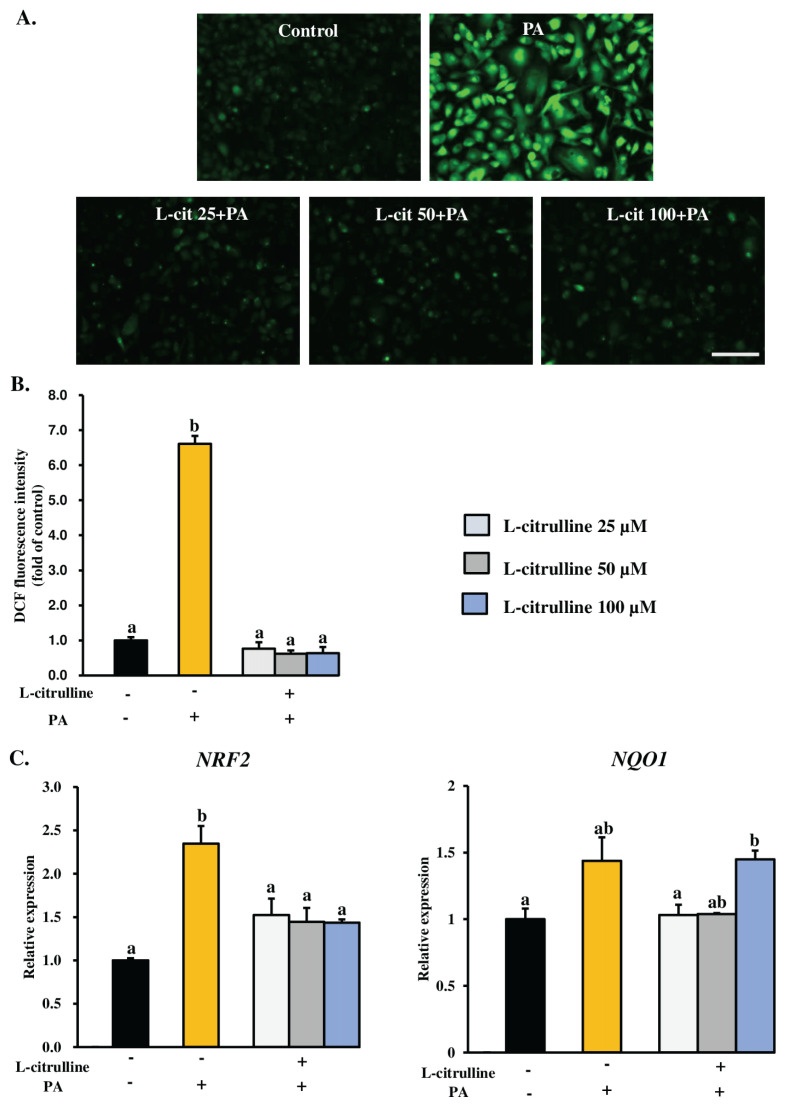
Effect of L-citrulline (L-cit) on intracellular ROS levels and oxidative stress-related gene expressions in MAC-T cells following PA treatment. Confluent cells (90% to 100%) were treated with 300 μM PA, either alone or with L-citrulline pretreatment at concentrations of 25, 50, and 100 μM, for 24 h. (A) Representative photomicrographs show DCF fluorescence in control cells, cells treated with PA alone, and cells co-treated with PA and different concentrations of L-citrulline. Scale bar = 200 μm. (B) The quantitative DCF fluorescence is based on the images in Figure 5A. The average fluorescence intensity was obtained from ten images for each experiment. The data from three independent experiments were combined, analyzed, and expressed as the fold change relative to control cells, presented as mean±SEM. (C) The relative mRNA expressions of *NRF2* and *NQO1* were determined by RT-qPCR and normalized to *ACTB* levels. The relative transcript expression was calculated using the 2^−ΔΔCt^ method and is presented as values relative to the control cells (received no L-citrulline and PA). The data, presented as mean±SEM, are based on three independent experiments. ROS, reactive oxygen species; MAC-T, mammary alveolar cell-T; PA, palmitic acid; DCF, dichlorofluorescein; SEM, standard error of the mean; RT-qPCR, real-time quantitative polymerase chain reaction; *NRF2*, nuclear factor (erythroid-derived 2)-like 2; *NQO1*, NAD(P)H quinone oxidoreductase 1; *ACTB*, β-actin. A Tukey’s honestly significant difference test was performed to compare the treatment means. ^a,b^ The means with uncommon letters differed significantly (p<0.05).

ROS are naturally produced during cellular metabolism, but excessive amounts can lead to cell death [15]. Given that PA is known for ROS generation [26,27], we analyzed intracellular ROS levels to determine whether L-citrulline pretreatment following PA treatment can reduce PA-induced ROS accumulation in MAC-T cells. As depicted in Figure 5A, L-citrulline pretreatment significantly decreased PA-induced intracellular ROS production. Additionally, the DCF fluorescence intensities in cells co-treated with 25, 50, and 100 μM L-citrulline and PA were 0.77±0.18, 0.62±0.10, and 0.63±0.18-fold of control, respectively, whereas PA-treated cells alone 6.61± 0.23-fold of control (Figure 5B).

As oxidative stress is mediated by ROS, we examined the mRNA expression of *NRF2* and its downstream signaling antioxidant gene, NAD(P)H quinone oxidoreductase 1 (*NQO1*), as marker of oxidative stress to determine whether L-citrulline pretreatment can alleviate PA-induced oxidative stress in MAC-T cells. The results showed that L-citrulline-treated cells significantly downregulated the PA-induced upregulation of *NRF2* mRNA expression, while *NQO1* expression remained nearly statistically similar (Figure 5C). *NRF2* is a key regulator of oxidative stress, and these findings suggest that L-citrulline mitigates PA-induced oxidative stress in MAC-T cells.

## DISCUSSION

During the transition period in cows, NEB triggers the release of stored fat, leading to a significant increase in the levels of free fatty acids (FFAs) in the blood [28]. Among these FFAs, PA is the most abundant saturated fatty acid. Although PA is an energy precursor, it can cause cellular dysfunction and apoptosis [29]. Previous studies have shown that PA induces severe ER stress and oxidative stress-mediated apoptosis in cells [8,13,14]. Therefore, it is crucial to understand the interactions between ER stress and oxidative stress during PA-induced cell death. Additionally, an effective supplementation strategy is essential to mitigate PA-induced cell death in MAC-T cells.

Our study demonstrated that treating MAC-T cells with 4-PBA (an ER stress inhibitor) significantly mitigated the PA-induced generation of ROS and the mRNA expression of *NRF2*, *ATF4*, and *CHOP*. A strong correlation was observed between the expression of ER stress markers (*ATF4* and *CHOP*) and ROS production in MAC-T cells, aligning with the findings of Zeeshan et al [30], who reported that the overexpression of ER stress markers *ATF4* and CHOP directly contributes to ROS production in the ER. According to Cao and Kaufman [31], protein misfolding in the ER induces ER stress, leading to oxidative stress and cell death. Yang et al [26] reported that 4-PBA significantly reduced PA-induced intracellular ROS generation and apoptosis in H9c2 cells. In our study, we observed a significant decrease in PA-induced intracellular ROS generation and oxidative stress in MAC-T cells by blocking ER stress with 4-PBA. These findings indicate that ER stress triggers oxidative stress during PA-induced MAC-T cell death.

Furthermore, we suppressed oxidative stress in cells through NAC treatment (acts as a ROS scavenger). We found that NAC, either alone or combined with PA, significantly reduced the PA-induced ROS production and mRNA expressions of *NRF2*, *ATF4*, and *CHOP*. This finding aligns with a previous report showing that NAC decreased PA-induced ER stress and apoptosis in H9c2 cells [26]. When cells encounter oxidative stress, the unfolded protein response may be activated, impeding the normal formation of disulfide bonds in the ER and inducing the expression of *CHOP* [15]. According to Zhang et al [32], the connection between ER stress and redox signaling in apoptosis is due to the accumulation of misfolded proteins resulting from excessive ROS, which triggers an ER stress-mediated apoptotic signal. In our study, we observed a significant decrease in the expression of ER stress markers *ATF4* and *CHOP* mRNA through blocking oxidative stress by NAC, suggesting that oxidative stress induces ER stress during PA-induced MAC-T cell apoptosis. Collectively, our data indicate that both ER and oxidative stresses interact with each other during PA-induced MAC-T cell death.

In this study, PA reduced the viability of MAC-T cells by 40%, and various concentrations of pretreated L-citrulline with PA treatment significantly increased cell viability. We used a fixed concentration of 300 μM of PA and exposed the cells for 24 h, according to Sharmin et al [33]. Compared to the control group, PA significantly reduces cell viability, which is consistent with the findings of our previous study [33]. However, our current study demonstrated that pretreatment with L-citrulline significantly rescued cell viability from PA-reduced cell viability in MAC-T cells.

In the present study, we observed a significant upregulation of *GRP78*, *ATF4*, and *CHOP* mRNA expression, as well as increased protein expression of phospho-PERK and cleaved caspase-3 in MAC-T cells treated with PA. Moreover, these increased mRNA and protein expressions were notably decreased with L-citrulline pretreatment (Figure 4A and 4B). ER dysfunction, in response to various stimuli, can lead to the accumulation of unfolded or misfolded proteins, thereby causing ER stress [31]. To counteract this, *GRP78* (an ER-resident molecular chaperone) detaches from the three stress-sensor transmembrane proteins, activating a homeostatic intracellular signaling network known as the unfolded protein response [34]. After induction of ER stress, *GRP78* expression increases sharply, suggesting its potential use as an ER stress indicator. In response to ER stress, the activated PERK suppresses general protein translation and promotes the translation of *ATF4* through the phosphorylation of *eIF-2α* [9]. In severe or persistent ER stress conditions, *ATF4* increases the transcription of *CHOP*, leading to cell apoptosis [10]. However, *CHOP* overexpression is required for ER stress-mediated cell death. Our previous studies also reported that PA induces severe ER stress-mediated apoptosis in MAC-T cells through increased mRNA expression of *ATF4* and *CHOP*, and protein expression of phospho-PERK and cleaved caspase-3 [8]. As L-citrulline pretreatment significantly decreased the PA-induced *GRP78*, *ATF4*, and *CHOP* mRNA expression, as well as phospho-PERK and cleaved caspase-3 protein expression, it potentially ameliorates the severe ER stress induced by PA in MAC-T cells.

Our results demonstrated that L-citrulline pretreatment significantly reduced PA-induced ROS levels. Mechanistically, ROS, the central oxygen-promoting oxide, is produced in excess in response to PA treatment, leading to oxidative stress, decrease in cell numbers, and increased apoptosis [35]. In this study, L-citrulline pretreatment downregulated the PA-induced upregulation of *NRF2* mRNA expression, a key regulator of oxidative stress. Under non-stress conditions, *NRF2* binds with Kelch-like ECH-associated protein 1 (Keap1) in the cytoplasm and is marked for degradation through a polyubiquitination-mediated proteasomal process, maintaining a lower level of NRF2 [36]. During oxidative stress, the sulfhydryl groups on Keap1 are oxidized, causing change in the confirmation of Keap1. Subsequently, *NRF2* is released and translocated into the nucleus, where it binds with the antioxidant response element and promotes the transcriptional activation of antioxidant genes like *NQO1* [36]. The increased expression of *NRF2* and *NQO1* by PA treatment is a self-defense mechanism, but it cannot reduce the PA-induced oxidative stress. However, L-citrulline can reduce the PA-induced oxidative stress in MAC-T cells. This aligns with the findings of Ham et al [19], who reported that L-citrulline prevents oxidative stress-induced C2C12 muscle cell wasting. Similarly, other studies have also shown that L-citrulline exhibits biological effects, including antioxidant, anti-inflammatory, and anticancer activity [37].

Our study has provided compelling evidence of the close relationship between ER and oxidative stresses, demonstrating how they synergistically exacerbate PA-induced MAC-T cell death. In this complex scenario, L-citrulline has emerged as a significant player, showing promise in modulating these stress-mediated cell deaths. The exact molecular mechanism by which L-citrulline ameliorates ER and oxidative stress-mediated PA-induced cell death remains to be fully understood. However, a substantial body of evidence suggests that L-citrulline enhances nitric oxide (NO) production by increasing the availability of L-arginine. This NO acts as a critical signaling molecule, regulating oxidative stress by scavenging ROS and enhancing the activity of antioxidant enzymes like superoxide dismutase and glutathione peroxidase [37,38]. These enzymes alleviate oxidative stress by reducing ROS levels, thereby protecting the ER from oxidative damage. Furthermore, L-citrulline supports the synthesis of glutathione (a vital cellular antioxidant) through its precursor role in the production of L-arginine [39]. Elevated levels of glutathione levels can shield the cells from damage caused by ROS produced during ER stress. Perturbation of either the *PERK* or *ATF4* pathway leads to an increase in the production of ER-derived ROS [40]. By enhancing glutathione levels, L-citrulline may reduce the accumulation of misfolded proteins, thus alleviating ER stress in MAC-T cells.

Overall, this study suggests that L-citrulline enhances PA-reduced cell viability and inhibits PA-induced ER and oxidative stresses, making it a promising option for mitigating the PA-induced MAC-T cell death. Therefore, dietary supplementation with L-citrulline could be beneficial for preserving the MEC numbers of lactating cows in the early stages of lactation to maintain optimal milk production.

In summary, the current study demonstrated that both ER stress and oxidative stress are key mechanisms underlying PA-induced MAC-T cell death. Moreover, this study shows that treating MAC-T cells with L-citrulline pretreatment enhances PA-reduced cell viability and inhibits PA-induced ROS generation, oxidative stress, and ER stress. Our *in vitro* study represents the initial evidence that L-citrulline treatment effectively shields MAC-T cells from PA-induced cell death by suppressing ER and oxidative stresses. Consequently, L-citrulline merges as a promising nutritional strategy for promoting sustainable milk production, addressing the negative energy balance challenges encountered during cows’ lactation period.

## Figures and Tables

**Table 1:** List of primers used for real-time quantitative polymerase chain reaction analysis

Gene	Orientation	Primer sequence (5′ to 3′)	GenBank accession no.	Reference
*ACTB*	Forward	CATCGCGGACAGGATGCAGAAA	NM_173979.3	[21]
	Reverse	CCTGCTTGCTGATCCACATCTGCT		
*CHOP*	Forward	CTGAAAGCAGAGCCTGATCC	NM_001078163.1	[21]
	Reverse	GTCCTCATACCAGGCTTCCA		
*ATF4*	Forward	CCGAGATGAGCTTTCTGAGC	NM_001034342	[21]
	Reverse	AGCATCCTCCTTGCTGTTGT		
*GRP78*	Forward	GATTGAAGTCACCTTTGAGATAGATGTG	XM_024998380.2	[22]
	Reverse	GATCTTATTTTTGTTGCCTGTACCTTT		
*NRF2*	Forward	CCAGCACAACACATACCA	AB162435.1	[22]
	Reverse	TAGCCGAAGAAACCTCATT		
*NQO1*	Forward	CAACAGACCAGCCAATCA	NM_001034535.1	[22]
	Reverse	ACCTCCCATCCTTTCCTC		

*ACTB*, β-actin; *CHOP*, C/EBP homologous protein; *ATF4*, activating transcription factor 4; *GRP78*, glucose-regulated protein 78; *NRF2*, nuclear factor (erythroid-derived 2)-like 2; *NQO1*, NAD(P)H quinone oxidoreductase 1.
